# Genomic Characterization of Papillary Thyroid Carcinoma: Age Differences in Tumor Aggressiveness and Immune Infiltration

**DOI:** 10.3390/diagnostics15232937

**Published:** 2025-11-21

**Authors:** Wei Ao, Shuqian Chen, Tenghong Liu, Bo Wang, Wenxin Zhao

**Affiliations:** 1 Department of Thyroid Surgery, Fujian Medical University Union Hospital, Fuzhou 350001, China; aow9060@gmail.com (W.A.); tenghong000608@163.com (T.L.); 2 Shengli Clinical Medical College, Fujian Medical University, Fuzhou 350001, China; 13960951567@163.com; 3 Fujian Clinical Research Center for Precision Management of Thyroid Cancers, Fuzhou 350001, China; 4 Division of Thyroid and Parathyroid Endocrine Surgery, Department of Otolaryngology-Head and Neck Surgery, Massachusetts Eye and Ear Infirmary, Harvard Medical School, Boston, MA 02114, USA

**Keywords:** age difference, papillary thyroid carcinoma, cervical lymph node metastasis, immune infiltration, genomic characterization

## Abstract

**Background:** Adolescents and young adults (AYA) with papillary thyroid carcinoma (PTC) often present with more extensive cervical lymph node metastasis (LNM) than older adults (AD). We aimed to identify age-associated molecular and immune features that might explain this phenotype and to explore potential translational implications for managing aggressive AYA PTC. **Methods:** We analyzed clinical and transcriptomic data from 501 PTC cases in The Cancer Genome Atlas (TCGA), stratified as AYA (<30 years, n = 64) and AD (≥30 years, n = 437). An institutional RNA-seq cohort (n = 13; 7 AYA, 6 AD) was used to screen for differentially expressed genes (DEGs). DEGs were defined by *p* ≤ 0.05 and |log2 fold change| ≥ 1. Intersection with invasion- and dissemination-related gene sets yielded a final age-related DEG list. Functional enrichment (GO/KEGG via DAVID), PPI network analysis (STRING, Cytoscape/cytoHubba), and immune deconvolution (CIBERSORT LM22) were performed. Protein-level validation was carried out by immunohistochemistry (IHC) in an independent cohort (n = 56; 28 AYA, 28 AD). Statistical comparisons used chi-square/Fisher’s exact tests for categorical variables, *t*-tests or nonparametric tests for continuous variables, and EdgeR with FDR correction for transcriptomic analyses. **Results:** In TCGA, LNM was more frequent in AYA than in AD (62.1% vs. 47.8%, *p* = 0.031). From intersected analyses, we identified 239 core DEGs distinguishing highly invasive, age-related tumors. Key upregulated genes in AYA included *CXCR4*, *OPCML* and *S100A2*; downregulated genes included *ATP1A3*, *CHL1*, *HLA-DRA* and *IL-1β*. Enriched pathways involved extracellular matrix organization, cell adhesion, calcium signaling and canonical oncogenic cascades (PI3K-Akt, MAPK, Wnt, Ras). Immune deconvolution showed reduced naïve B cells, M1 and M2 macrophages and resting mast cells and an increased proportion of M0 macrophages in AYA tumors. IHC validated differential protein expression for seven markers. Collectively, the data indicate an immune-suppressed, immune-excluded microenvironment in AYA PTC. **Conclusions:** AYA PTC exhibits distinct molecular and immune features that may underlie its propensity for lymphatic dissemination. These findings support evaluation of translational strategies, such as CXCR4 inhibition, restoration of antigen presentation, and macrophage reprogramming, to convert “cold” tumors into immune-permissive lesions. Validation in larger, prospective, multicenter cohorts is required.

## 1. Introduction

Thyroid cancer is the most common malignancy of the endocrine system. According to statistics from the National Cancer Institute (NCI) [1], the incidence of thyroid cancer among American women in 2024 was approximately 3.35%, which is three times that observed in men, making it the eighth most common cancer among women. The overall incidence of thyroid cancer has been declining by about 2% per year since 2014. This trend may be attributed to the avoidance of routine screening in asymptomatic populations and the increasingly stringent criteria for fine-needle aspiration biopsy. In contrast, the incidence among adolescents aged 15–19 has been rising by approximately 4–5% annually since 1998, with the rate in females being roughly five times higher than in males. Moreover, studies [2] have shown that, among newly diagnosed cancers in adolescents and young adults, thyroid cancer has consistently ranked first over the years. There has been growing attention on the distinct tumor characteristics observed in AYA patients with PTC.

PTC is the most common histological subtype of thyroid cancer, accounting for 80–90% of all cases [3]. Patients with PTC who undergo standardized surgical procedures combined with thyroid-stimulating hormone (TSH) suppression therapy and postoperative radioactive iodine treatment (such as I^131^) typically achieve a 5-year survival rate exceeding 90%. However, PTC patients who experience recurrence, metastasis, or exhibit iodine-refractory characteristics continue to present significant challenges in clinical management. Adolescents and young adults (AYA), defined in this study as individuals younger than 30 years [4], have attracted increasing research attention. Compared to their older counterparts, AYA patients generally exhibit smaller tumor sizes and a more favorable prognosis; nevertheless, they often manifest more aggressive tumor biology [5,6], including higher rates of lymph node metastasis and recurrence.

Previous studies [6] have hypothesized that adolescent and young adult (AYA) patients with PTC tend to lack coexisting mutations in telomerase reverse transcriptase (TERT) and tumor protein p53 (TP53). These genetic alterations that are commonly associated with more aggressive disease and poorer prognosis in adult (AD) PTC. This molecular distinction may partially explain why the AYA population often presents with a high rate of lymph node metastasis yet generally has a more favorable prognosis. However, the underlying molecular mechanisms driving these differences remain unclear, and the clinical challenges of high lymph node metastasis and recurrence persist. Recent advances in high-throughput sequencing technologies, such as transcriptome sequencing, have provided novel insights into the molecular landscapes of various cancers [5,7,8]. These technologies can identify key genetic alterations and signaling pathways that drive tumor progression, offering powerful tools to uncover age-specific molecular signatures and improve our understanding of the distinct clinical behavior observed in AYA PTC. Meanwhile, the development of novel targeted therapies and drug delivery strategies, such as antibody drug conjugates (ADCs), has progressed rapidly for recurrent, metastatic, or iodine refractory PTC. However, clinical research and practices specifically focused on PTC in the AYA population remain scarce. More comprehensive molecular profiling of papillary thyroid carcinoma in AYA patients is urgently needed to identify novel, effective therapeutic targets and improve outcomes for this underserved population.

Since the FDA’s approval of the first immune checkpoint inhibitor targeting CTLA-4 in 2011, immunotherapy has rapidly advanced, with an increasing number of agents targeting various immune checkpoints entering clinical practice in recent years. Many cancer patients have benefited from these therapies. However, response rates across most solid tumors remain modest, typically ranging from 15% to 40% [9]. Therefore, it is of considerable interest to investigate the composition of the immune microenvironment through transcriptomic data to determine whether the AYA PTC population, characterized by a high rate of lymph node metastasis, might represent a subgroup that could benefit from immunotherapy.

In this study, we hypothesized that AYA PTC harbors distinct differentially expressed genes (DEGs) and signaling pathways that may account for the observed differences in clinical behavior. To test this, we performed RNA sequencing on PTC samples obtained from both AYA and adult (AD) patients to identify DEGs. These findings were further validated using immunohistochemistry. Our objectives were to: (1) characterize the transcriptomic and molecular differences between AYA and adult PTC; (2) explore key biological processes and signaling pathways associated with age-specific clinical behavior; and (3) identify critical DEGs involved in these mechanisms. By elucidating these molecular distinctions, we aim to provide a more refined understanding of PTC in the AYA population, ultimately contributing to improved clinical management and patient outcomes.

## 2. Materials and Methods

### 2.1. Patient Selection and Clinical Data

#### 2.1.1. TCGA Cohort

Clinical and transcriptomic data were accessed from The Cancer Genome Atlas (TCGA) for 501 papillary thyroid carcinoma (PTC) patients. Patients were stratified into Adolescents and Young Adults (AYA, <30 years, n = 64) and Older Adults (AD, ≥30 years, n = 437) groups based on age at diagnosis. Data included demographics, tumor characteristics, and lymph node metastasis (LNM) status, adhering to TCGA study protocols.

#### 2.1.2. Institutional Cohort

An additional cohort of 13 PTC patients was recruited from Fujian Medical University Union Hospital for transcriptomic analysis. These patients were categorized as AYA (n = 7) or AD (n = 6). Clinical characteristics were documented, including age, sex, tumor focality (unifocal vs. multifocal), extrathyroidal extension (ETE), and LNM. Formalin-fixed, paraffin-embedded (FFPE) tissue blocks from an independent validation cohort of 56 PTC patients were used for immunohistochemical verification. These patients were classified as AYA (n = 28) or AD (n = 28). The study involving human participants was reviewed and approved by the Ethics Committee of Fujian Medical University Union Hospital (Approval No. 2024KY151, approved on 19 July 2024). Written informed consent was obtained from all participants prior to sample collection. All procedures were conducted in accordance with relevant ethical guidelines and institutional regulations, and personal data were handled in compliance with institutional privacy policies. Additionally, all tumor specimens included in this study were of the Classic type of PTC.

### 2.2. Transcriptome Analysis

#### 2.2.1. RNA Extraction and Sequencing

Tumor and adjacent normal thyroid tissues were collected from the institutional cohort, snap-frozen in liquid nitrogen, and stored at −80 °C until RNA extraction. Total RNA was extracted using TRIzol reagent (Invitrogen, Carlsbad, CA, USA) according to the manufacturer’s protocol. RNA concentration and purity were measured using a NanoDrop spectrophotometer (Thermo Fisher Scientific, Waltham, MA, USA). RNA integrity was assessed with an Agilent 2100 Bioanalyzer (Agilent Technologies, Santa Clara, CA, USA). RNA integrity was evaluated using the RNA Integrity Number (RIN), and samples with RIN values greater than 6.5 were considered acceptable. A total of 1 µg of RNA was used for library construction. RNA-seq libraries were prepared using the NEBNext^®^ Ultra™ Directional RNA Library Prep Kit for Illumina (New England Biolabs, Ipswich, MA, USA). mRNA was isolated using oligo(dT) beads, fragmented, and reverse-transcribed to cDNA. Second-strand synthesis, end repair, adapter ligation, and PCR amplification were performed to generate sequencing libraries, which were sequenced on an Illumina NovaSeq 6000 platform with a paired-end 150 bp (PE150) strategy at Novogene (Beijing, China) [10].

#### 2.2.2. Differential Gene Expression Analysis

After normalization of the transcriptomic data, we performed four binary differential-expression comparisons: tumor versus adjacent normal tissue (TUMOR vs. NORMAL), adolescents and young adults versus adults (AYA vs. AD), invasion-related cancer versus non-invasion (IC vs. NIC), and dissemination-related cancer versus non-dissemination (DC vs. NDC). Group membership and grouping criteria are defined as follows and were determined from the clinical annotation provided in Supplementary Table S1 (columns “Age Group (AYA/AD)”, “primary neoplasm focus type”, and “extrathyroid carcinoma present extension status”): Age groups (AYA vs. AD): patients were classified by age at diagnosis as AYA (<30 years) or AD (≥30 years). The AYA group comprised samples AYA1–AYA7; the AD group comprised samples AD1–AD6. Invasion groups (IC vs. NIC): classification of invasion was based on the “extrathyroid carcinoma present extension status” annotation (presence vs. absence of extrathyroidal extension). The IC (invasion) group included AYA1, AYA2, AYA3, AD2, AD3, AD4, AD5, and AD6; the NIC (non-invasion) group included AYA4, AYA5, AYA6, AYA7, and AD1. Dissemination groups (DC vs. NDC): dissemination was defined using the “primary neoplasm focus type” annotation (multifocal versus unifocal as recorded in the clinical table). The DC (dissemination/multifocal) group included AYA1, AYA2, AYA4, and AYA5; the NDC (non-dissemination/unifocal) group included AYA3, AYA6, AYA7, AD1, AD2, AD3, AD4, AD5, and AD6. All group assignments and the sample-to-group mapping are provided in Supplementary Table S1. Genes were considered significantly differentially expressed if they met thresholds of *p* ≤ 0.05 and |log2 fold change| ≥ 1. These comparisons produced four gene sets: genes differentially expressed in cancer versus normal tissues (TUMOR vs. NORMAL), age-related genes (AYA vs. AD), dissemination-related genes (DC vs. NDC), and invasion-related genes (IC vs. NIC). To generate the age-related tumor DEG set used for downstream analyses, we intersected the TUMOR vs. NORMAL gene set with the age-related genes. These genes were visualized using the pheatmap package (version 1.0.12), which shows agglomerative hierarchical clustering of genes and samples, highlighting distinct gene expression patterns. Specifically, the algorithm calculates pairwise distances between genes based on their relative expression levels and a selected distance metric. Through iterative computation of these distances, genes are progressively grouped based on their similarity. Ultimately, genes are assigned to different subclusters according to their relative distances, thereby achieving effective clustering.

#### 2.2.3. Functional Enrichment Analysis

To understand the functional implications of the differentially expressed genes (DEGs), we performed Gene Ontology (GO) and Kyoto Encyclopedia of Genes and Genomes (KEGG) pathway enrichment analyses using DAVID (Database for Annotation, Visualization, and Integrated Discovery). The age-related tumor DEG set was intersected separately with the dissemination-related and invasion-related gene lists, yielding two intersection-derived gene sets (gene set 1 and gene set 2). The union of the two intersection-derived subsets constituted the final set of differentially expressed genes for further analysis. These DEGs were uploaded to DAVID and analyzed for enrichment in GO terms, including Biological Processes (BP), Molecular Functions (MF), and Cellular Components (CC), as well as KEGG pathways. Enriched GO terms and KEGG pathways were identified using DAVID’s functional annotation chart, with results visualized to show gene ratios and significance levels. This analysis provided insights into key biological processes, molecular functions, cellular components, and pathways linked to the aggressive clinical phenotype in age-related tumor tissues.

#### 2.2.4. Correlation and Core Gene Analysis

We conducted a correlation analysis of core DEGs associated with highly invasive, age-related tumors, displaying their Pearson correlation coefficients in a heatmap. We first created protein–protein interaction (PPI) networks using the STRING database (https://string-db.org/) for gene set 1 and gene set 2, respectively. We then used Cytoscape (version 3.9.1) and its cytoHubba plugin to analyze the top 30 core genes related to high invasion and multifocal dissemination from each set. The intersection of these genes resulted in core DEGs associated with highly invasive, age-related tumors. These core genes were further analyzed for gene-gene correlations using the corrplot package in R (version 0.90).

### 2.3. Immune-Cell Infiltration Analysis

The CIBERSORT algorithm [11] was used to estimate the immune cell infiltration from the RNA-seq data. For this analysis, normalized gene expression data from the RNA-seq experiments were input into the CIBERSORT algorithm using the LM22 gene signature matrix, which defines the expression profiles of 22 distinct leukocyte subsets. The output included the relative abundance of each immune cell type in each sample. The results were visualized to compare the immune cell composition between the AYA and AD groups, highlighting significant differences in the immune landscape of PTC tumors across different age groups, using the nonparametric Wilcoxon rank-sum test.

### 2.4. Immunohistochemistry (IHC) and Immunohistochemical Score (H-Score)

To maintain consistency, we selected the largest tumor focus in multifocal cases for sequencing and IHC. Formalin-fixed, paraffin-embedded specimens were cut into 4 μm sections. Slides were deparaffinized in xylene, rehydrated through a graded ethanol series, and subjected to antigen retrieval in citrate buffer (pH 6.0) using microwave heating. The primary antibodies used for IHC included Anti-HLA-DR (Abcam, Cambridge, UK, ab92511), Anti-ATP1A3 (Abcam, ab2826), Anti-OPCML (Abcam, ab238143), Anti-CXCR4 (Abcam, ab124824), Anti-IL-1*β* (Abcam, ab156791), Anti-S100A2 (Abcam, ab109494) and Anti-CHL1 (Abcam, ab106269). After primary antibody incubation and washes, sections were processed with the Elivision™ Plus IHC detection system (ready-to-use; Cat. No. kit9903; Maixin Biotech, Fuzhou, China). Per the manufacturer’s protocol, slides were incubated with the kit amplifier/enhancer for 20 min at room temperature (≈25 °C), followed by incubation with the HRP-conjugated secondary reagent for 30 min at room temperature. Immunoreactivity was visualized with 3,3′-diaminobenzidine (DAB). Following DAB development, nuclei were counterstained with hematoxylin, differentiated and blued in ammonia water, and coverslips were mounted with neutral resin.

Immunostained slides were independently reviewed by two senior pathologists (≥5 years’ experience) at the Department of Pathology, Fujian Medical University Union Hospital, who were blinded to all clinical information. Staining was scored semi-quantitatively based on the percentage of positive cells (0 = 0%; 1 = 1–24%; 2 = 25–49%; 3 = 50–74%; 4 = 75–100%) and staining intensity (0 = negative; 1 = weak; 2 = moderate; 3 = strong). The final H-score was obtained by multiplying the proportion score by the intensity score [12,13].

### 2.5. Statistical Analysis

Comparative analyses between AYA and AD groups were performed using chi-square tests or Fisher’s exact tests for categorical variables and *t*-tests or Mann–Whitney U tests for continuous variables. Statistical significance was set at a *p*-value < 0.05. Differentially expressed genes (DEGs) were analyzed using EdgeR with false discovery rate (FDR) correction for multiple testing. Hypergeometric tests were used for Gene Ontology (GO) and Kyoto Encyclopedia of Genes and Genomes (KEGG) pathway enrichment analysis, with *p*-values adjusted using Benjamini–Hochberg correction for multiple comparisons. All statistical analyses and data visualizations were performed using R (version 4.0.5) with the ggplot2 (version 3.3.3), pheatmap (version 1.0.12), clusterProfiler (version 3.18.1), and EdgeR (version 3.42.0) packages.

## 3. Results

### 3.1. Clinical Characteristics

#### 3.1.1. TCGA Cohort

The TCGA cohort included 501 papillary thyroid carcinoma (PTC) patients, stratified into Adolescents and Young Adults (AYA, <30 years, n = 64) and Older Adults (AD, ≥30 years, n = 437). Key clinical characteristics are summarized in Table 1. The majority of patients were female (73.3%). There was a higher prevalence of multifocal tumors in the AD group (47.6%) compared to the AYA group (38.0%) (Table 2).

Lymph node metastasis was more frequently observed in the AYA group (62.1%) compared to the AD group (47.8%), as represented by the N stage (*p* = 0.031). In the AD group, tumors were relatively larger and more frequently presented at advanced T stages (*p* < 0.001), illustrating the AYA phenotype of smaller primary lesions coupled with higher lymph node metastasis rates.

#### 3.1.2. Institutional Cohort

An additional cohort of 13 PTC patients was recruited from Fujian Medical University Union Hospital, categorized into AYA (n = 7) and AD (n = 6), as shown in Supplementary Table S1 for details. The mean age was 23.6 years for AYA and 52.8 years for AD. Key clinical characteristics are summarized in Table 3. Tumor size was larger in AYA (2.22 ± 0.58 cm vs. 1.68 ± 0.76 cm in AD), and a higher incidence of intrathyroidal spread was noted in AYA (5/7 vs. 1/6 in AD) (Table 3).

### 3.2. Differential Gene Expression

#### 3.2.1. Differential Gene Expression Analysis

**Figure 1:**
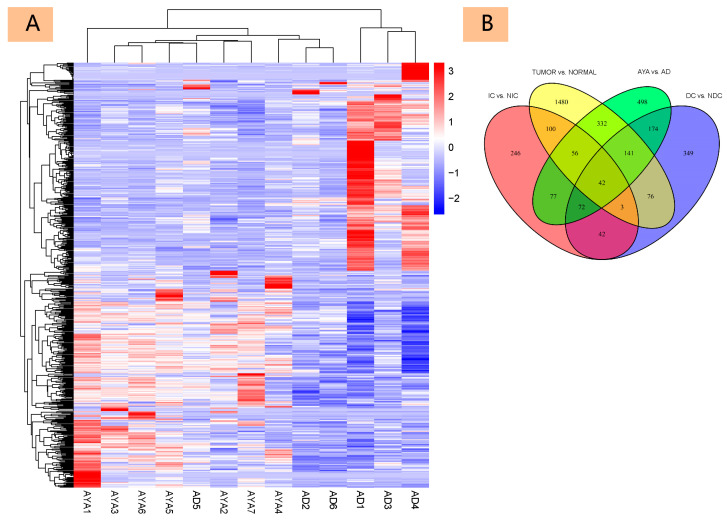
Differential Gene Expression Analysis in Age-Related Tumors. (**A**): Heatmap displaying the expression levels of 571 genes obtained by intersecting DEGs between papillary thyroid carcinoma (PTC) tissues and adjacent normal tissues with age-related DEGs. (**B**): Venn diagram illustrating the overlap between four sets of differentially expressed genes (DEGs): genes differentially expressed in cancer versus normal tissues, age-related genes, dissemination-related genes, and invasion-related genes.

A total of 571 genes were identified, representing the intersection of genes differentially expressed between PTC tissues and adjacent tissues (TUMOR vs. NORMAL) and genes differentially expressed between age groups (AYA vs. AD). This resultant set was visualized using a heatmap (Figure 1A), highlighting the distinct cancer gene expression patterns between the AYA and AD groups. A Venn diagram (Figure 1B) illustrates the intersections and connectivity among the four core gene sets: genes differentially expressed in cancer versus normal tissues, age-related genes, dissemination-related genes, and invasion-related genes.

#### 3.2.2. Functional Enrichment Analysis

**Figure 2:**
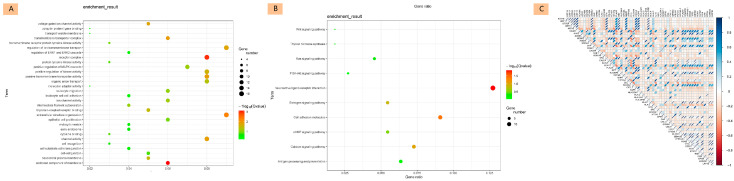
Functional Enrichment and Core Gene Analysis in Age-Related High Invasiveness Tumors. (**A**): Gene Ontology (GO) enrichment analysis of DEGs in age-related, highly invasive tumors. (**B**): Kyoto Encyclopedia of Genes and Genomes (KEGG) pathway enrichment analysis of DEGs. (**C**): Correlation heatmap showing the expression levels between 55 core genes associated with highly invasive and dissemination-related age-related tumors.

We intersected the 571 age-related DEGs with genes associated with invasion and tumor dissemination, resulting in two gene sets of 98 and 183 genes, respectively. Combining these sets, we obtained 239 DEGs of particular interest. Gene Ontology (GO) and Kyoto Encyclopedia of Genes and Genomes (KEGG) pathway enrichment analyses were conducted to understand the functional implications of the differentially expressed genes (DEGs). These analyses identified key biological processes, molecular functions, cellular components, and pathways enriched in highly invasive age-related tumor tissues (Figure 2A,B).

GO enrichment analysis highlighted several important processes, including extracellular matrix organization, regulation of ion transmembrane transport, leukocyte migration and adhesion, positive regulation of the MAPK cascade, and regulation of ERK1 and ERK2 cascades. Additionally, key molecular functions identified included G-protein coupled receptor binding, protein tyrosine kinase activity, and ubiquitin protein ligase binding.

KEGG pathway enrichment analysis revealed significant pathways such as cell adhesion molecules, calcium signaling pathway, antigen processing and presentation, estrogen signaling pathway, thyroid hormone synthesis, and well-known pathways related to tumor migration and invasion such as the PI3K-Akt signaling pathway, Wnt signaling pathway, and Ras signaling pathway. These findings emphasize the importance of cell adhesion, ion transmembrane transport, and cell signal transduction in the tumor invasion process, providing insights into potential mechanisms and therapeutic targets.

#### 3.2.3. Correlation and Core Gene Analysis

We first created protein–protein interaction (PPI) networks using the STRING database for the two gene sets of 183 and 98 genes, respectively. We then used Cytoscape and its cytoHubba plugin to analyze the top 30 core genes related to high invasion and multifocal dissemination from each set. The intersection of these genes resulted in core DEGs associated with highly invasive, age-related tumors. Correlation analysis of 55 core DEGs associated with highly invasive and dissemination-related age-related tumors was performed. A heatmap (Figure 2C) displayed the Pearson correlation coefficients of these genes. Cytoscape with its cytoHubba plugin was employed to analyze the top 30 core genes related to high invasion and multifocal dissemination.

#### 3.2.4. Differential Expression of Core Genes

Expression differences for seven core genes (*ATP1A3*, *CHL1*, *HLA-DRA*, *IL1B*, *CXCR4*, *OPCML*, and *S100A2*) between the AYA and AD groups are summarized in Table 4. The table reports log2 fold-change values and corresponding *p*-values for each gene; several genes show statistically significant age-related differential expression (exact values are provided in Table 4). These findings reflect distinct transcriptional profiles between the two age groups (statistical methods are described in the Section 2).

### 3.3. Immune-Cell Infiltration Analysis

**Figure 3:**
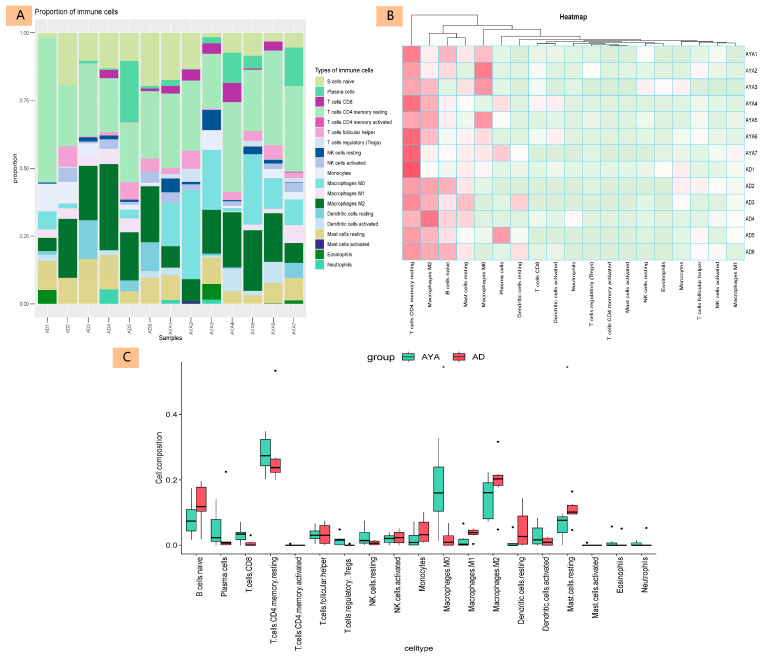
Analysis of Immune Cell Composition in Different Age Groups. This figure presents the analysis of immune cell composition between different age groups. (**A**) shows the relative abundance of various immune cell types in sequenced samples. (**B**) is a heatmap that displays the relative abundance of 19 immune cell types across 13 tumor samples, with color intensity ranging from low (light green) to high (dark red), representing the proportion of each cell type. The clustering analysis at the top reveals the similarity in immune cell distribution characteristics. (**C**) is a boxplot illustrating the differences in various immune cell types between the Adolescent and Young Adult (AYA, green) group and the Adult (AD, red) group in papillary thyroid carcinoma patients. Asterisks indicate the statistical significance of differences between the groups.

The immune microenvironment of AYA patients with PTC may exhibit significant immunosuppression and enhanced immune evasion characteristics. We analyzed immune cell infiltration from two perspectives: immune cell proportion (Figure 3A) and immune cell composition similarity (Figure 3B) across different groups and samples. The group difference analysis (Figure 3C) revealed that naive B cells, M1 and M2 macrophages, and resting mast cells were lower in AYA patients, while M0 macrophages were higher in the AYA group.

### 3.4. Immunohistochemistry (IHC)

**Figure 4:**
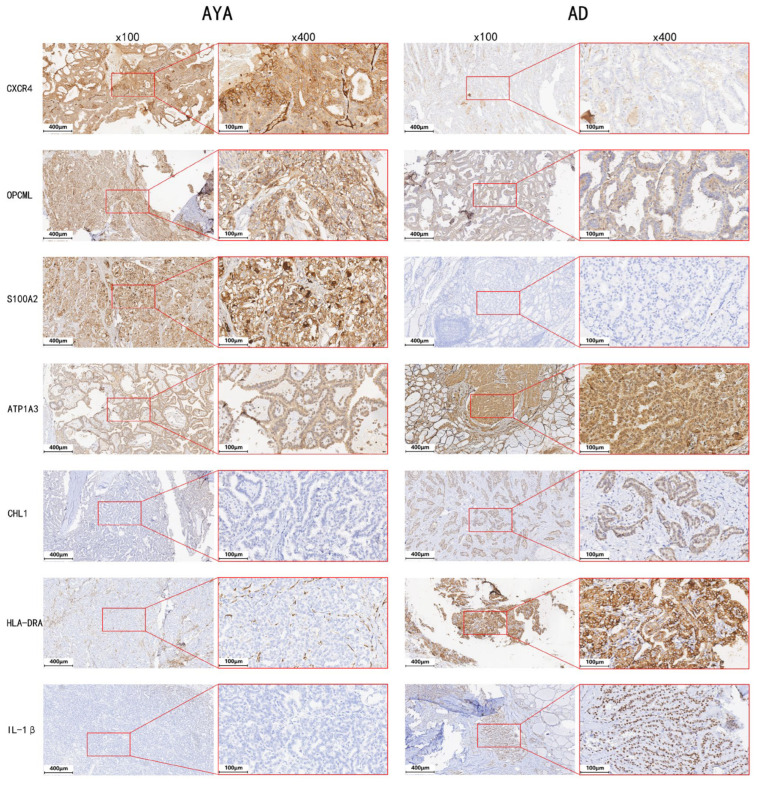
Immunohistochemical validation of key differentially expressed proteins in Adolescents and Young Adults (AYA) and Adults (AD). Immunohistochemical analysis showing the protein expression of key differentially expressed genes in papillary thyroid carcinoma (PTC). Representative images are now provided at both low-power (×100) and high-power (×400) magnification (scale bars shown in each panel) to allow assessment of staining at the tissue and cellular levels. Overall, CXCR4, S100A2, and OPCML show higher expression in AYA tumors, whereas ATP1A3, CHL1, HLA-DRA, and IL-1*β* exhibit lower expression compared with the AD group.

IHC analysis confirmed the protein-level differences in key differentially expressed genes. Representative images for seven markers are shown in Figure 4, demonstrating distinct expression patterns between AYA and AD patients. Compared with AD tumors, AYA tumors showed higher expression of *CXCR4* (*p* = 0.040), *S100A2* (*p* = 0.037), and *OPCML* (*p* = 0.038), and lower expression of *IL-1β* (*p* < 0.001), *CHL1* (*p* = 0.027), *ATP1A3* (*p* = 0.048), and *HLA-DRA* (*p* = 0.026). Statistical comparisons were performed using independent-samples *t*-tests.

## 4. Discussion

Adolescents and Young Adults with PTC exhibit higher risks of lymph node metastasis (LNM) compared to Adult patients. In our transcriptomic comparisons between these age groups, we identified marked molecular differences. Functional enrichment analysis of age-associated differentially expressed genes (DEGs) in AYA patients with PTC revealed significant enrichment in pathways related to cell adhesion, ion transmembrane transport, and cell signal transduction. These pathways are likely crucial in promoting tumor invasiveness. Guided by these results, we prioritized a subset of candidate genes for further study. Notably, AYA tumors showed upregulation of *CXCR4*, *OPCML*, and *S100A2* and downregulation of *ATP1A3*, *CHL1*, *HLA-DRA*, and *IL-1β*. *IL-1β*, *CXCR4*, and *HLA-DRA* are particularly associated with immune cell infiltration. Tumors from AYA patients exhibited a pronounced immunosuppressive environment, indicative of impaired anti-tumor immune responses, thereby reinforcing the importance for performing a thorough cervical lymph node dissection at the time of initial surgery in this population. These findings underscore the importance of characterizing age-specific molecular alterations in AYA PTC. A deeper understanding of these distinctive mechanisms is essential for developing personalized management strategies for AYA patients, for example, preoperative molecular subtyping of the primary tumor by core-needle biopsy to assess the appropriate extent of cervical lymph node dissection, and to enable more accurate postoperative risk stratification for recurrence. This perspective aligns with prior cohort studies that reported factors associated with lymph node metastasis in AYA PTC patients [14].

An age cutoff of 30 years was chosen to divide patients into the 2 groups for two complementary reasons, although NCI commonly defines the adolescent and young adult (AYA) population as 15–39 years of age. First, there are prior epidemiologic precedents that support a younger cutoff: several studies have employed a 30-year boundary to distinguish subgroups with divergent epidemiologic and molecular characteristics (for example, Lee et al. in osteosarcoma [15]). From the perspective of age-stratified disease burden, a report from the Children’s Cancer Institute (American University of Beirut Medical Center) noted that for epithelial cancers including cervical, breast, lung, and gastrointestinal, the DALY burden rises sharply after age 29, arguing for separate evaluation of patients <30 years in outcome and intervention studies [4]. Second, our own molecular data provide internal validation for this choice: unsupervised clustering (Figure 1A) revealed that the majority of cases aged <30 formed a coherent molecular cluster distinct from older adults, whereas a single 38-year-old “AD1” case clustered with the adult group, likely reflecting its proximity to middle age. This observation led us to adopt 30 years of age as our cutoff threshold.

In the cluster analysis of our samples (Figure 1A), we observed an intriguing phenomenon: The heatmap shows clustering of most AD and AYA samples, except for AD5, which clusters with the AYA group. To investigate this outlier, we performed a detailed examination of its gene expression profile relative to the main AD and AYA cohorts. After confirming the validity of the sequencing data, our primary clinical consideration focused on AD5’s Hashimoto’s history: AD5 is the only AD sample with a documented history of Hashimoto’s thyroiditis (HT), importantly, the AYA sample most closely clustered with AD5 is also an HT patient. We therefore hypothesize that differences in bulk tissue composition (increased immune- and stromal-cell content in Hashimoto samples) contribute to AD5’s proximity to the AYA cluster. Further molecular investigation confirmed this hypothesis by identifying the genes driving this separation. We analyzed the top 30 hub genes from a Protein–Protein Interaction (PPI) network of the 571 clustered genes. Relative to the other AD samples, both AD5 and the AYA group exhibited higher expression of genes associated with aggressive tumor features and inflammation (e.g., CXCL2, TUBB3, RRM2, and MMP1) and lower expression of differentiation markers and metastatic regulators (e.g., KIT and NCAM1). These findings suggest that the AD5 patient, despite being older than the AYA cohort, possesses a tumor that shares key molecular characteristics, a signature of inflammation, loss of differentiation, and increased invasiveness, which aligns more closely with the AYA group.

The PI3K-Akt, MAPK, Wnt, and Ras signaling pathways were particularly enriched, indicating that these genes might drive tumor migration and invasion through these channels. RET/PTC rearrangements, common in thyroid cancer, can activate both the MAPK and PI3K-Akt pathways, leading to tumor progression and aggressiveness. Studies [16] have shown that the transcriptional effects of CCDC6-RET rearrangements are primarily due to the CCDC6-RET Y451F mutation. Cancer cells with the CCDC6-RET Y451F mutation exhibit strong invasive and migratory properties while maintaining low proliferation rates similar to normal thyroid cells [17]. Further transcriptional analysis revealed that CCDC6-RET Y451F mutant strains exhibit lower IL-1β and higher CXCR4 expression compared to normal thyroid cells, consistent with our findings. GO and KEGG enrichment analyses suggest the importance of the calcium signaling pathway in tumor migration and invasion. *S100A2*, a key molecule regulating intracellular calcium balance, was upregulated in the AYA group [18]. *S100A2* plays a specific role in the progression of papillary thyroid carcinoma. Research by Ito [19] found that in two types of cancer originating from thyroid follicular cells, most cases of papillary carcinoma were *S100A2* positive, while all cases of follicular carcinoma were negative. Previous clinical studies [20] also reported significant differences in *S100A2* RNA and protein levels in lymph node metastases of papillary thyroid carcinoma, consistent with our study. Moreover, the higher the degree of tumor dedifferentiation (higher invasiveness), the higher the expression of *S100A2*, such as in anaplastic carcinoma, where *S100A2* expression is higher than in papillary carcinoma. In summary, *S100A2* represents a promising target for antibody–drug conjugate (ADC) development in aggressive PTC.

This study’s results align with existing literature suggesting that AYA patients with PTC exhibit different invasive behaviors compared to AD patients. Previous studies have indicated that younger patients often present with more aggressive disease despite generally favorable long-term outcomes [21]. Our findings support these observations by providing a detailed molecular analysis that highlights specific genes and pathways involved in age-specific tumor behaviors. For example, *CXCR4*, highly expressed in this group. It may facilitate thyroid cancer migration and invasion through multiple pathways, including epithelial–mesenchymal transition (EMT), cancer stem cell induction and maintenance, angiogenesis, and new blood vessel formation [22]. Clinical studies have shown that *CXCR4* expression is positively correlated with more invasive and poorly prognostic tumors in thyroid cancer [23]. The finding of elevated CXCR4 in the AYA group echoes reports implicating CXCR4 in tumor invasiveness, suggesting it may also be a promising therapeutic target for intervention in papillary thyroid carcinoma. Targeting *CXCR4* and its related pathways could be a potential therapeutic strategy for managing aggressive PTC in AYA patients. Specifically, a number of *CXCR4* inhibitors are already in early-phase clinical development for various cancers [24]. For example, agents such as AMD3100 and AMD3465 have been evaluated in breast and bladder cancers, AMD11070 in melanoma and oral cancer, MSX-122 in breast cancer, head and neck squamous cell carcinoma, and uveal melanoma, and CTCE-9908 in prostate cancer. Nevertheless, these targeted therapies still face certain limitations that warrant careful consideration. On one hand, these agents are currently being explored for their efficacy in other tumor types; on the other hand, the molecular mechanisms of *CXCR4* in thyroid cancer remain to be elucidated, necessitating further in-depth studies in the future.

*OPCML* is a highly conserved glycoprotein closely associated with cell adhesion. In various tumors, *OPCML* acts as a putative tumor suppressor gene (TSG) [25]. In papillary thyroid carcinoma, *OPCML* expression is higher in the AYA group, potentially due to hypermethylation of the promoter region CpG. In the TCGA thyroid cancer database, thyroid cancer samples showed higher expression levels compared to corresponding normal thyroid adjacent tissues, with expression levels decreasing with age, consistent with our findings [26]. Similarly, *CHL1* has been reported as a tumor suppressor gene in multiple cancer-related studies [27]. *CHL1* expression is dysregulated in major epithelial malignancies such as ovarian cancer and esophageal squamous cell carcinoma. Reduced *CHL1* expression is associated with poor differentiation, increased invasion, lymph node metastasis, advanced tumor stage, and decreased overall survival.

Previous studies have demonstrated a strong association between *ATP1A3* expression and the progression of glioma [28,29] and ovarian cancer [30]. We observed low expression of *ATP1A3* and its correlation with increased KRAS signaling and other oncogenic pathways aligns with evidence showing the importance of Na^+^/K^+^-ATPase in tumor progression [28]. However, not all PTC cases progress through KRAS-dominant pathways, which may partly explain the variability in *ATP1A3* expression among patients.

In summary, these insights emphasize the necessity of understanding age-specific molecular and clinical characteristics in PTC for developing personalized diagnostic, therapeutic, and prognostic strategies. These molecular profiles in AYA patients underscore enhanced cell migration, diminished cell adhesion, and altered extracellular matrix interactions, contributing to the aggressive tumor characteristics observed in this age group. Understanding these mechanisms is crucial for developing targeted therapies to improve outcomes for AYA patients, including precise diagnostics, personalized treatments, and accurate prognostic evaluations.

The immune microenvironment in AYA patients with PTC is characterized by strong immunosuppression and enhanced immune evasion, as evidenced by reduced levels of naive B cells, M1/M2 macrophages, and resting mast cells, alongside an increase in M0 macrophages, This pattern is consistent with previous studies [31,32,33], indicating that AYA patients may have a poorer potential response to tumor immunotherapy. The immunosuppressive environment in AYA patients may limit the effectiveness of standard immunotherapies, such as checkpoint inhibitors, which rely on an active and coordinated immune response. For AYA patients, precise preoperative lymph node assessment and thorough lymph node dissection could potentially improve clinical outcomes. Understanding the distinct immune dynamics in AYA patients with PTC is crucial for developing more effective and personalized treatment strategies [34]. The consistent reduction in M1 and M2 macrophages and the increase in M0 macrophages suggest an inhibited or regulated immune system in AYA patients. This could be due to chronic inflammatory conditions, such as autoimmune thyroid diseases (AITD), leading to impaired or delayed macrophage differentiation. Additionally, tumor-secreted cytokines like IL-10 and TGF-β may inhibit typical macrophage activation and differentiation. The decrease in M2 macrophages in highly invasive tumors with lymph node metastasis could reflect a more complex immune regulatory network, enhancing tumor escape mechanisms and supporting tumor survival and dissemination.

Translationally, our findings suggest several strategies that may help convert the immunologically “cold” AYA PTC microenvironment into one more responsive to immunotherapy. First, the consistent upregulation of CXCR4 supports testing CXCR4 antagonists, either alone or in combination with PD-1/PD-L1 inhibitors or with intratumoral innate immune activators such as STING or TLR agonists. This approach may reduce tumor spread and improve immune-cell infiltration. Second, the downregulation of HLA class II genes, including HLA-DRA, indicates impaired antigen presentation. Short-term IFN-γ exposure or carefully dosed epigenetic modulators could be explored to restore antigen-presentation capacity and enhance T-cell recognition, although the risk of autoimmune activation must be considered. Third, the increase in M0 macrophages suggests a block in macrophage differentiation. Approaches that target tumor-associated macrophages, such as CSF1R inhibition, CD40 agonists, or TLR/STING agonists, may promote M1 polarization and strengthen innate antitumor activity. All of these strategies require mechanistic validation in appropriate in vitro co-culture systems and in vivo models, with careful monitoring for immune-related toxicity given the high background prevalence of autoimmune thyroid disease in the AYA population.

In this study, we identified differentially expressed genes (DEGs) with potential biological significance between AYA PTC patients with high cervical lymph node metastasis and adult (AD) PTC patients, and we further delineated a series of molecular markers worthy of additional investigation through multi-level analysis. However, several limitations remain. First, regrettably, due to initial oversights in our study design and prevailing financial constraints (funding issues), we were unable to secure a strictly N-stage-matched cohort. The sample size of our RNA sequencing cohort was relatively small (n = 7 for the AYA group and n = 6 for the AD group), and only one case in the AYA group was ≤14 years old. This limitation may compromise the generalizability and statistical robustness of our findings. Given tumor heterogeneity, the small cohort may underestimate or overestimate the differences in the expression of certain genes. Our study was therefore designed to serve as a preliminary screening and target-identification effort focused on capturing the molecular characteristics of the clinically observed aggressive biological phenotype (e.g., ETE, dissemination) in AYA patients, and we believe the findings, despite these limitations, warrant further dedicated investigation. Second, the retrospective nature of the sample collection introduces the possibility of selection and recall biases, and the single-center design further restricts the external validity of our conclusions. Although the credibility of our results was enhanced by immunohistochemical (IHC) validation of the corresponding protein expression levels in these samples, future research should aim to validate key molecular markers in larger, prospective, multicenter cohorts that include patients across different age groups to ensure the robustness and translational potential of the findings. Moreover, the molecular mechanisms underlying the high rate of cervical lymph node metastasis in AYA patients, the therapeutic value of the identified DEGs, and their dynamic changes during PTC progression require further elucidation through longitudinal studies and functional assays. Addressing these gaps will help advance the precision classification of PTC and the development of individualized treatment strategies.

## 5. Conclusions

The AYA group shows significant clinical differences in PTC compared to the AD group, with higher risks of lymph node metastasis (LNM). RNA sequencing results revealed significant differences in gene expression, with key genes (upregulated *CXCR4*, *OPCML*, and *S100A2*, and downregulated *ATP1A3*, *CHL1*, *HLA-DRA*, and *IL-1β*) associated with tumor invasiveness. Pathway analysis indicated significant enrichment in pathways related to cell adhesion, ion transmembrane transport, and cell signal transduction in the AYA group. Immune cell analysis shows an immunosuppressive environment in AYA tumors, with decreased naive B cells, M1/M2 macrophages, and resting mast cells, and increased M0 macrophages, suggesting a poorer response to standard immunotherapies and the need for targeted strategies. Incorporating age-specific molecular markers into clinical management can improve diagnostic accuracy and help develop personalized treatment strategies for AYA patients [7,35]. Future research should validate these findings in larger cohorts and explore the therapeutic potential of these markers.

## Figures and Tables

**Table 1:** Clinical characteristics of the TCGA cohort.

Characteristic	Number of Cases (%)
Age (years)	
<30 years	64 (12.8%)
≥30 years	437 (87.2%)
Gender	
Male	134 (26.7%)
Female	367 (73.3%)
Number of Foci	
Unifocal	264 (53.8%)
Multifocal	227 (46.2%)
Extra-thyroidal Extension (ETE)	
None	333 (68.9%)
Minimal (T3)	131 (27.1%)
Moderate/Advanced (T4a)	18 (3.7%)
Very Advanced (T4b)	1 (0.2%)
Lymph Node Metastasis	
None	168 (43.5%)
Present	218 (56.5%)
T Stage	
T1/2	308 (61.7%)
T3/4	191 (38.3%)
N Stage	
N0	226 (50.1%)
N1	225 (49.9%)
M Stage	
M0	281 (96.9%)
M1	9 (3.1%)
Cancer Stage	
Stage I/II	331 (66.3%)
Stage III/IV	168 (33.7%)

**Table 2:** Comparison of clinicopathological features between Adolescents and Young Adults (AYA) and Adults (AD) in the TCGA cohort. Data for continuous variables are presented as mean ± standard deviation (SD), while categorical variables are presented as frequency (n) or percentage (%), unless otherwise specified.

Characteristic	Adolescents and Young Adults (Age < 30 years)	Adults (Age ≥ 30 years)	*p*-Value
Gender			0.522
Male	15 (23.4%)	119 (27.2%)	
Female	49 (76.6%)	318 (72.8%)	
Extra-thyroidal Extension (ETE)			0.049
None	50 (79.4%)	283 (67.4%)	
Minimal (T3)	12 (19.0%)	119 (28.3%)	
Moderate/Advanced (T4a)	1 (1.6%)	17 (4.0%)	
Very Advanced (T4b)	0 (0%)	1 (0.2%)	
Maximum Tumor Diameter (cm)	2.75 ± 1.16	3.04 ± 1.69	0.222
Number of Lymph Node Metastases	5.00 ± 8.03	3.70 ± 6.36	0.246
Lymph Node Metastasis			0.132
None	20 (34.5%)	148 (45.1%)	
Present	38 (65.5%)	180 (54.9%)	
Number of Foci			0.134
Unifocal	44 (62.0%)	220 (52.4%)	
Multifocal	27 (38.0%)	200 (47.6%)	
T Stage			<0.001
T1/2	52 (72.2%)	256 (60.0%)	
T3/4	20 (27.8%)	171 (40.0%)	
N Stage			0.031
N0	25 (37.9%)	201 (52.2%)	
N1	41 (62.1%)	184 (47.8%)	
M Stage			0.954
M0	33 (97.1%)	248 (96.9%)	
M1	1 (3.0%)	8 (3.1%)	
Cancer Stage			<0.001
Stage I/II	73 (100%)	258 (60.6%)	
Stage III/IV	0 (0%)	168 (39.4%)	
Clinical Outcomes			
Follow-Up Time (months)	44.43 ± 31.42	40.09 ± 32.84	0.326
OS (Overall Survival) (months)	42.82 ± 31.59	39.13 ± 32.60	0.389
DFI (Disease-Free Interval) (months)	40.58 ± 31.04	39.03 ± 33.03	0.745

**Table 3:** Clinical characteristics of the institutional cohort. Data for continuous variables are presented as mean ± standard deviation (SD), while categorical variables are presented as frequency (n) or percentage (%), unless otherwise specified.

Characteristic	Adolescents and Young Adults (Age < 30 years)	Adults (Age ≥ 30 years)
Number of Patients	7	6
Gender		
Male	2	1
Female	5	5
Age (years)		
Mean Age	23.6	52.8
Age Range	9–29	38–66
Preoperative Blood Parameters		
TSH (μIU/mL)	1.99 ± 0.54	4.00 ± 2.83
FT3 (pmol/L)	5.51 ± 0.68	5.22 ± 0.68
FT4 (pmol/L)	11.15 ± 2.07	10.97 ± 1.47
TG (ng/mL)	89.2 ± 173.7	118.3 ± 180.4
TGAb (IU/mL)	0.6 ± 1.0	12.1 ± 29.5
TPOAb (IU/mL)	4.4 ± 9.4	1.0 ± 0.7
Preoperative Ultrasound Evaluation		
Tumor Size (cm)	2.22 ± 0.58	1.68 ± 0.76
Tumor Characteristics		
Cystic/Solid	Solid: 5, Cystic: 2	Solid: 5, Cystic: 1
Invasion/Intrathyroidal Spread/Hashimoto’s		
Invasion	3/7	5/6
Intrathyroidal Spread	4/7	0/6
Hashimoto’s	3/7	1/6
Lymph Node Metastasis		
Central Compartment	7/7	5/6
Lateral Neck	6/7	3/6
TNM Staging		
T1/2	7	2
T3/4	0	4
N0	0	0
N1a	1	3
N1b	6	3
M0	7	6
M1	0	0
Cancer Stage		
Stage I/II	7	3
Stage III/IV	0	3

**Table 4:** Key differentially expressed genes with log2 fold change values and *p*-values.

Gene	Description	log2 Fold Change (AYA vs. AD)	*p*-Value
CXCR4	C-X-C Motif Chemokine Receptor 4	1.26	0.003
S100A2	S100 calcium binding protein A2	1.88	0.008
OPCML	Opioid Binding Protein/Cell Adhesion Molecule-Like	4.90	<0.001
IL-1*β*	Interleukin-1*β*	−1.82	0.021
CHL1	Cell adhesion molecule L1-like	−3.13	<0.001
ATP1A3	ATPase Na^+^/K^+^ transporting subunit alpha 3	−7.24	<0.001
HLA-DRA	Major Histocompatibility Complex, Class II, DR Alpha	−9.86	<0.001

## Data Availability

Gene expression data with Affymetrix chips are publicly available in the National Center for Biotechnology Information Gene Expression Omnibus database (NCBI GEO accession no. GSE275666).

## Supplementary Materials

The following supporting information can be downloaded at: https://www.mdpi.com/article/10.3390/diagnostics15232937/s1, Supplementary Table S1: Detailed Clinical Characteristics of the Institutional Cohort of 13 Patients with Papillary Thyroid Carcinoma.

## Abbreviations

The following abbreviations are used in this manuscript:

AYA	adolescents and young adults
PTC	papillary thyroid carcinoma
AD	adults
TCGA	The Cancer Genome Atlas
DEGs	differentially expressed genes
IHC	immunohistochemistry
NCI	National Cancer Institute
TSH	thyroid-stimulating hormone
TERT	telomerase reverse transcriptase
TP53	tumor protein p53
ADCs	antibody drug conjugates
LNM	lymph node metastasis
ETE	extrathyroidal extension
FFPE	formalin-fixed, paraffin-embedded
RIN	RNA Integrity Number
IC	Invasion-related Cancer
NIC	Non-Invasion-related Cancer
DC	Dissemination-related Cancer
NDC	Non-Dissemination-related Cancer
GO	Gene Ontology
KEGG	Kyoto Encyclopedia of Genes and Genomes
BP	Biological Processes
MF	Molecular Functions
CC	Cellular Components
PPI	protein–protein interaction
EMT	epithelial–mesenchymal transition
TSG	tumor suppressor gene
AITD	autoimmune thyroid diseases
